# Ciprofloxacin and moxifloxacin could interact with SARS-CoV-2 protease: preliminary in silico analysis

**DOI:** 10.1007/s43440-020-00169-0

**Published:** 2020-10-15

**Authors:** Krzysztof Marciniec, Artur Beberok, Paweł Pęcak, Stanisław Boryczka, Dorota Wrześniok

**Affiliations:** 1 Department of Organic Chemistry, Faculty of Pharmaceutical Sciences in Sosnowiec, Medical University of Silesia, Jagiellońska 4, 41-200 Sosnowiec, Poland; 2 Department of Pharmaceutical Chemistry, Faculty of Pharmaceutical Sciences in Sosnowiec, Medical University of Silesia, Jagiellońska 4, 41-200 Sosnowiec, Poland

**Keywords:** Ciprofloxacin, Moxifloxacin, COVID-19 Main Protease (M^pro^), Molecular docking

## Abstract

**Background:**

A large body of research has focused on fluoroquinolones. It was shown that this class of synthetic antibiotics could possess antiviral activity as a broad range of anti-infective activities. Based on these findings, we have undertaken in silico molecular docking study to demonstrate, for the first time, the principle for the potential evidence pointing ciprofloxacin and moxifloxacin ability to interact with COVID-19 Main Protease.

**Methods:**

In silico molecular docking and molecular dynamics techniques were applied to assess the potential for ciprofloxacin and moxifloxacin interaction with COVID-19 Main Protease (M^pro^). Chloroquine and nelfinavir were used as positive controls.

**Results:**

We revealed that the tested antibiotics exert strong capacity for binding to COVID-19 Main Protease (M^pro^). According to the results obtained from the GOLD docking program, ciprofloxacin and moxifloxacin bind to the protein active site more strongly than the native ligand. When comparing with positive controls, a detailed analysis of the ligand–protein interactions shows that the tested fluoroquinolones exert a greater number of protein interactions than chloroquine and nelfinavir. Moreover, lower binding energy values obtained from *K*_DEEP_ program were stated when compared to nelfinavir.

**Conclusions:**

Here, we have demonstrated for the first time that ciprofloxacin and moxifloxacin may interact with COVID-19 Main Protease (M^pro^).

## Introduction

In recent years, several fluoroquinolone derivatives were synthesized and approved by FDA as a broad spectrum, antibacterial agents used in the treatment of respiratory and urinary tract infections [1, 2]. These drugs are effective in the treatment of the hospital-acquired infections in which resistance to older antibacterial classes is suspected [3]. Their mechanism of action is based on inhibition of the activities of prokaryotic DNA gyrase–topoisomerase II and topoisomerase IV which are involved in replication, transcription and DNA synthesis [1].

Some commercially available fluoroquinolones (e.g. ciprofloxacin) used for the treatment of bacterial infections were shown to be active against other non-bacterial incidents. Fluoroquinolones may have antiviral (e.g. vaccinia virus, papovavirus, human cytomegalovirus, herpes simplex virus types 1 and 2, hepatitis C virus) [4–6], antifungal, and antiparasitic actions at the clinically achievable concentrations. This broad range of anti-infective activities is due to one common mode of action: the inhibition of type II topoisomerases or inhibition of viral helicases [7]. Therefore, the respiratory fluoroquinolones could be considered as an adjunct treatment in COVID-19 [8].

Coronaviruses (CoVs) can infect humans and vertebrate animals. CoV infections affect the respiratory, digestive, liver, and central nervous systems of humans and animals [9]. The new strain of CoV was identified at the end of 2019, named 2019-nCoV, and emerged during an outbreak in Wuhan, China [10]. No specific therapies for COVID-19 are currently available [11]. Proteases represent potential targets for the inhibition of CoV replication, and the protein sequences of the SARS-CoV M^pro^ and the 2019-nCoV M^pro^ (also known as 3-chymotrypsin-like protease—3CL^pro^) are 96% identical, and the active sites in both proteins remain free from mutations [12]. According to the crystallographic data, amino acids His 41, His 164, Met 49, Met 165, Thr 190, and Gly 143 play an important role in the stabilization the ligand–M^pro^ complexes [13, 14]. Because the proteases play a key role in viral replication, they are considered as molecular targets when developing antiviral drugs [15, 16]. What is important, the development of medicines treating diseases caused by SARS-CoV-2; the fastest way is to find potential agent among the already-marketed drugs.

A series of actions have been taken to control the epidemic of the 2019-nCoV virus, and the effective therapeutic methods are in urgent needs to prevent infection. Due to the time-consuming process of developing new medicines, drug repositioning may be the only solution to overcome infectious diseases. There are no data demonstrating the possible interaction of fluoroquinolones with COVID-19 Main Protease (M^pro^). Therefore, to provide a basis for potential evidence indicating the fluoroquinolones ability to interact with COVID-19 protease, the present experimental study was designed to investigate the binding capacity of ciprofloxacin and moxifloxacin to the COVID-19 target protein. The in silico molecular docking technique was applied to check if the two already-marketed fluoroquinolones derivatives may interact with the virus main protease.

## Materials and methods

The three-dimensional (3D) structures of studied compounds were generated in their low-energy conformation using Gaussian 16 (revision A.03) computer code [17] at the density functional theory (DFT, B3LYP) and 6–311 + G(d,p) basis sets. Calculations were performed using the X-ray coordinates of ciprofloxacin, moxifloxacin and chloroquine as the input structure obtained from the Cambridge Crystallographic Data Centre (CCDC ID: NUWFUI, ABABIQ, and CDMQUI, respectively).

Target macromolecule for molecular docking studies was obtained from the Protein Data Bank (https://www.rcsb.org/). We used 3D crystal structures of COVID-19 main protein (PDB ID: 5R7Z).

Genetic Optimization for Ligand Docking (GOLD) 5.6.3 [18] was used for the docking analysis. The Hermes visualiser in the GOLD Suite was used to further prepare receptors. All hydrogen atoms, including those necessary to define the correct ionisation and tautomeric states of residues such as Asp, Glu and His, were added and all water molecules and ligands were deleted for docking. GOLD is an automated ligand docking program that uses a genetic algorithm to explore the full range of ligand conformational flexibility with partial flexibility of the protein (flexibility of receptor hydrogens) [19]. The region of interest used for GOLD docking was defined as all the COVID-19 protein residues within the 6 Å of the reference ligand. Default values of all other parameters were used and the complexes were submitted to 100 genetic algorithm runs using the GoldScore fitness function. After calculations, only the ten highest scored pose was returned as a docking result for ligand-cavity configuration. All obtained results were ranked according to their score value and presented in GOLD arbitrary units (a.u.).

Calculation of protein–ligand binding free energy was performed using *K*_DEEP_ predictor based on DCNNs (https://playmolecule.org/Kdeep) [20].

Molecular docking details were visualized using the BIOVIA Discovery Studio virtual environment [21].

Molecular dynamics simulation was performed with Nanoscale Molecular Dynamics software ver. 2.13 (NAMD, https://www.ks.uiuc.edu/Research/namd/) [22]. All input files were prepared using QwikMD [23] computer program based on GOLD output complexes. Protein–ligand systems have been solvated with 0.15 mol/L NaCl water box. Then system was minimized, annealed and equilibrated. After that, 10 ns production simulation was performed. Results were analyzed using Visual Molecular Dynamics package (VMD, https://www.ks.uiuc.edu/Research/vmd/) [24].

## Results

COVID-19 main protease (M^pro^) structure was obtained from PDB (PDB ID: 5R7Z). The native ligand for 5R7Z is *N*-[2-(5-fluoranyl-1*H*-indol-3-yl)ethyl]ethanamide (HWH). To validate the accuracy of GoldScore protocol in GOLD, the cocrystallized COVID-19 M^pro^ protein reference ligand was redocked into the binding site of protein. The root-mean-square deviation (RMSD) value between crystallized structure and docking pose of control is 0.4201, which shows a good accuracy in the docking simulation by GoldScore protocol. So, we employ GoldScore protocol as suitable for ciprofloxacin and moxifloxacin docking with COVID-19 protein. We used the zwitterionic state of ciprofloxacin and moxifloxacin in our calculations. The tested compounds ranked by GOLD are shown in Table 1. All obtained results were presented in GOLD arbitrary units (a.u.) Highest scores in silico correspond to a strong binding affinity, and the most probable ligand–protein system in vivo.

**Table 1:** Scoring functions of tested compounds

Compound name	Docking Score (a.u.)	Binding energy (kcal/mol)
HWH	42.84	− 6.45
Ciprofloxacin	50.16	− 8.05
Moxifloxacin	51.39	− 8.66
Chloroquine	58.12	− 8.13
Nelfinavir	60.41	− 7.50

**Fig. 1:**
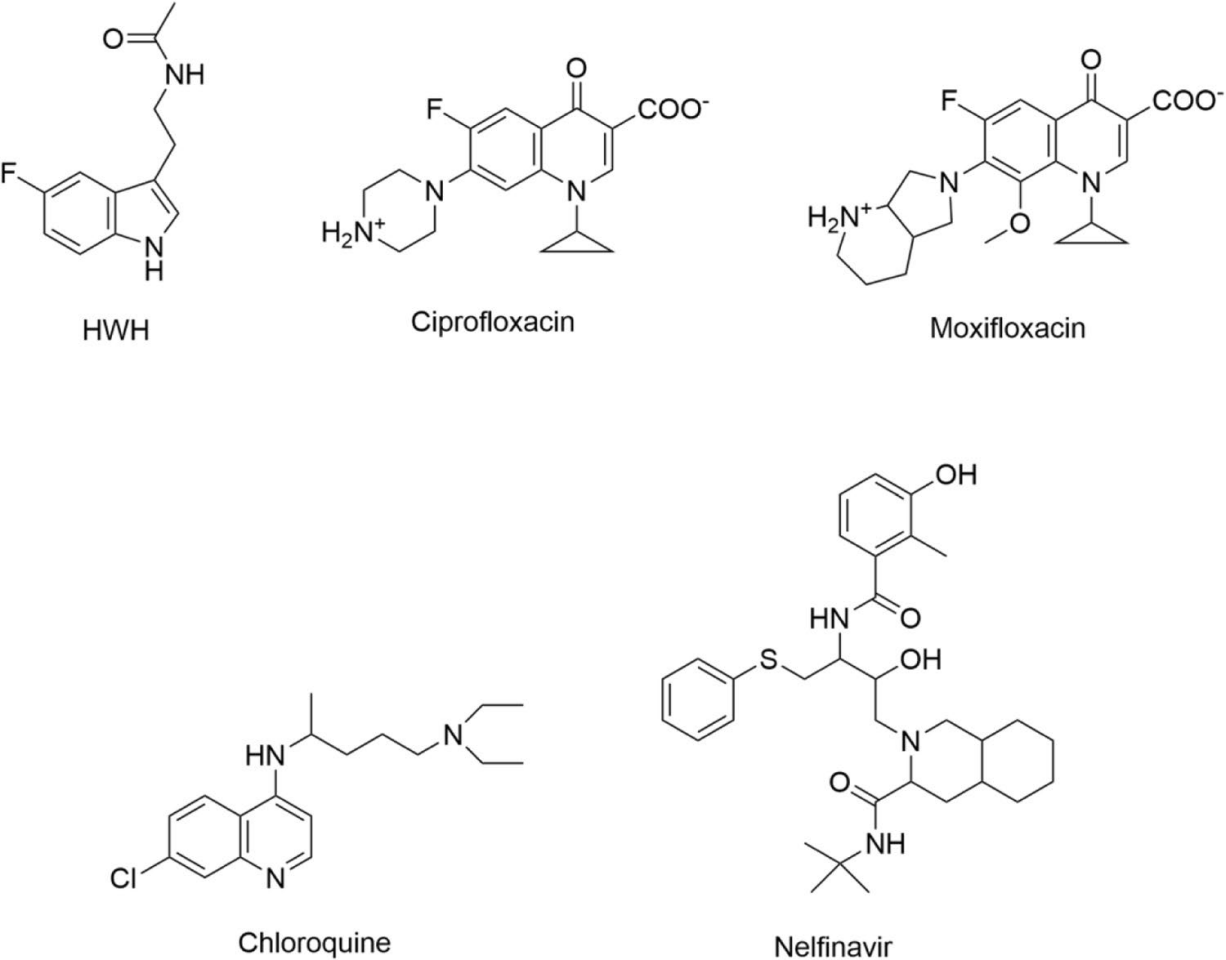
Structure of compounds used in this study

Accurately predicting protein–ligand binding affinities is an important problem in computational chemistry since it can substantially accelerate drug discovery for virtual screening. In this work, we also used a fast machine-learning approach for predicting binding affinities using state-of-the-art deep convolutional neural networks (DCNNs). For comparison and validation of docking results, we used *K*_DEEP_ predictor. *K*_DEEP_ predicts binding affinities using DCNNs and calculates the binding energy Δ*G* [kcal/mol] of protein–ligand complexes. In this case, the more negative the Δ*G* value of the binding reaction, the higher the binding affinity of the ligand for its specific target protein (Fig. 1).

Based on the simulations performed in GOLD, ciprofloxacin and moxifloxacin were found to be more strongly associated with the active protein site than the reference ligand HWH, but less strongly than chloroquine and nelfinavir (Table 1).

The analysis of the results obtained in *K*_DEEP_ show that that moxifloxacin showed the lowest binding energy value compared to reference ligands. The tested compounds demonstrate a degree of fit in the following order: moxifloxacin > chloroquine > ciprofloxacin > nelfinavir > HWH (Table 1).

**Fig. 2:**
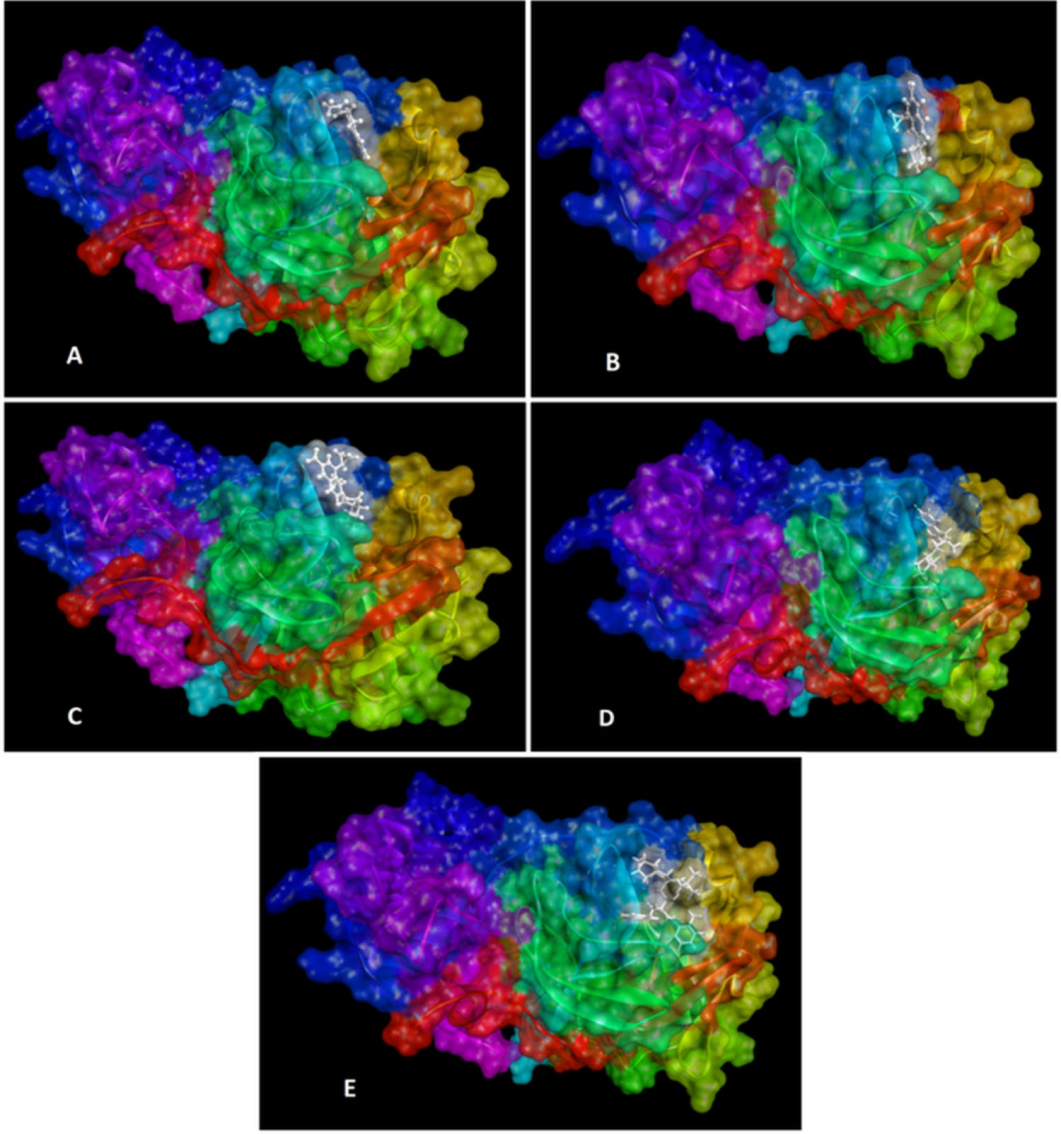
Docking pose of COVID-19 M^pro^ protein complex with HWH (**a**), ciprofloxacin (**b**), moxifloxacin (**c**), chloroquine (**d**) and nelfinavir (**e**)

**Fig. 3:**
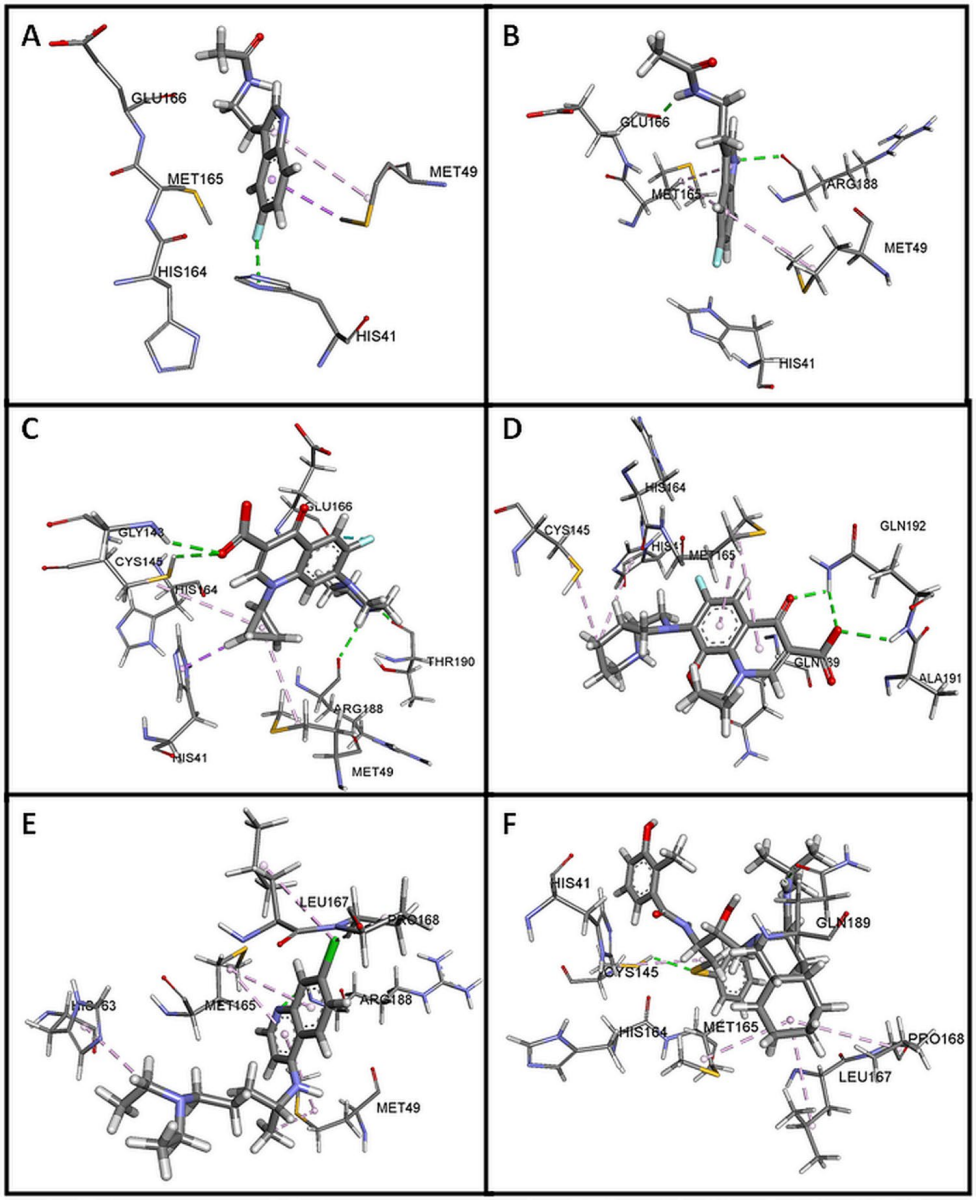
The visualization of hydrogen bonds (green) and hydrophobic interaction (violet and yellow) between HWH (**a** in crystal structure and **b** redocked**)**, ciprofloxacin (**c**), moxifloxacin (**d**), chloroquine (**e**) and nelfinavir (**f**) with COVID-19 M^pro^

**Fig. 4:**
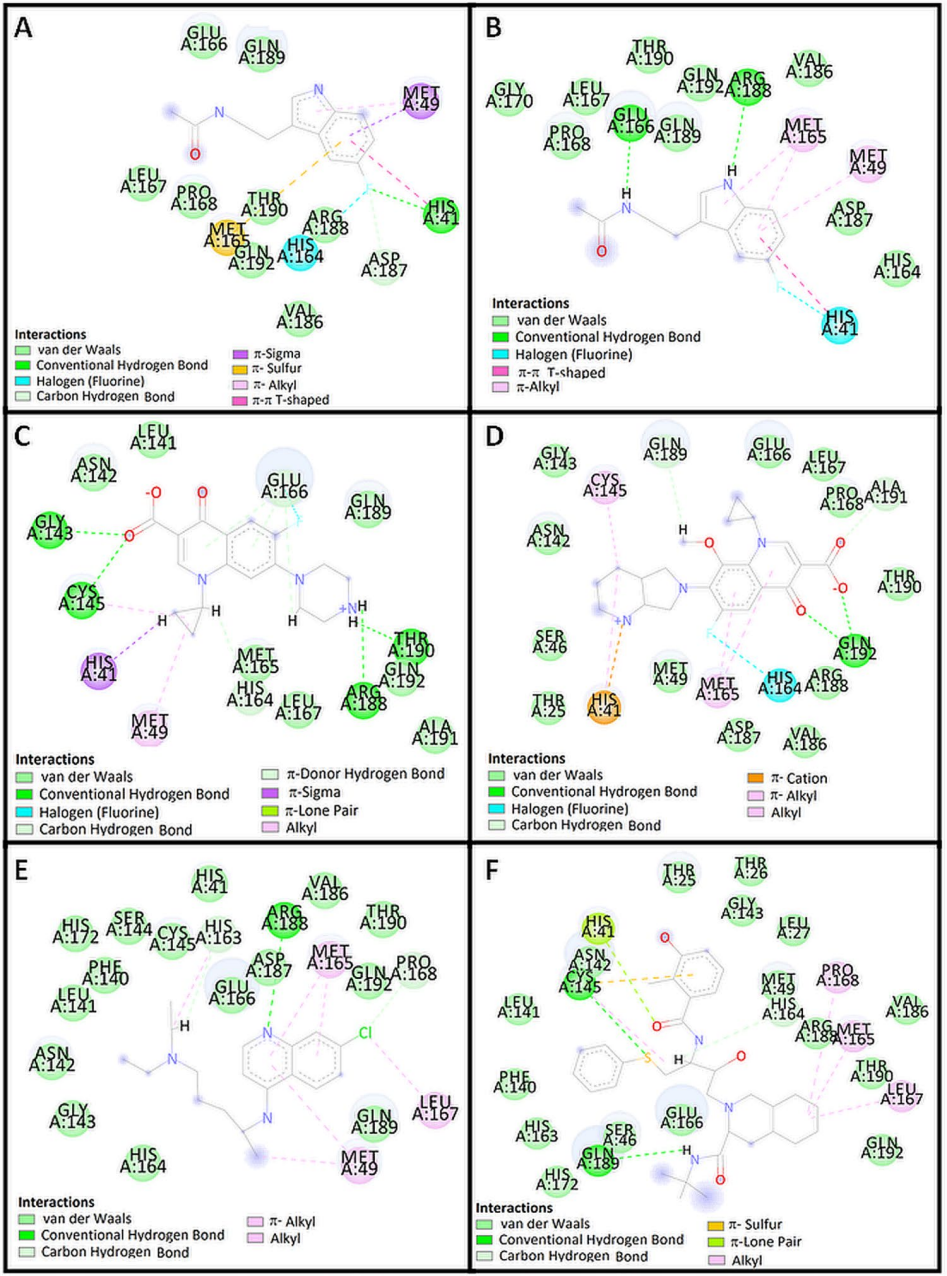
Binding 2D model of HWH (**a** in crystal structure and **b** redocked) and predicted binding model of ciprofloxacin (**c**), moxifloxacin (**d**), chloroquine (**e**) and nelfinavir (**f**) with COVID-19 M^pro^

The indole moiety of HWH was located deep in the matrix of the active site near the side chains of the residues in positions **Met 49, Met 165, His 41 **and **His 164** (Figs. 2a, 3a, 4a). Complex of WHW with protein revealed that the fluorine-substituted benzene ring of indole moiety forms hydrogen bond with **His 41**. Another dipolar interaction between fluorine and the amide group of **His 164** is also visible, as well as hydrophobic interaction involving an aromatic or aliphatic carbon or sulfur in the receptor and an aromatic carbon in the ligand. These interactions are also present in re-docked pose of HWH (Figs. 3b, 4b).

According to the docking poses in Figs. 3c, 4c, carboxylate group of ciprofloxacin can interact to form hydrogen bonds with residues Gly 143 and Cys 145 of the protein. Complex of ciprofloxacin with 5R7Z revealed that the piperazine moiety forms another hydrogen bonds between a positively charged nitrogen of piperazine and Arg 188 and Thr 190. In addition, numerous hydrophobic interactions (including interaction with **His 41**, **Met 49** and **His 164**) influence the increase in the stability of the complex (Table 2).

**Table 2:** Interaction of tested compounds with COVID-19 M^pro^

Protein		Ligand		Interaction	
Name	Residue	Name	Residue	Type	Distance (Å)
COVID-19 M^pro^ (5R7Z)	His 41 Glu166 His 164 Met 49 Met 165 His 41 Met 49	HWH	Fluorine Ethyl Fluorine Benzene ring Benzene ring Benzene ring Pyrole ring	Conventional hydrogen bond Carbon hydrogen bond Dipole–dipole π–sigma π–sulfur π–π, T-shape π–alkyl	2.98 3.17 3.64 3.56 5.67 4.93 5.00
	Gly 143 Cys 145 Arg 188 Thr 190 His 164 Glu 166 Glu 166 Glu 166 Glu 166 His 41 Glu 166 Cys 145 Met 49	Ciprofloxacin	Carboxylate Carboxylate Piperazine Piperazine Cyclopropyl Ethyl Fluorine Pyridone ring Benzene ring Cyclopropyl Benzene ring Cyclopropyl Cyclopropyl	Conventional hydrogen bond Conventional hydrogen bond Conventional hydrogen bond Conventional hydrogen bond Carbon hydrogen bond Carbon hydrogen bond Dipole–dipole π–donor hydrogen bond π–donor hydrogen bond π–sigma π–lone pair Alkyl–alkyl Alkyl–alkyl	2.57 2.67 2.27 1.51 2.31 2.31 2.46 3.09 2.52 2.38 2.92 5.46 4.89
	Gln 192 Gln 192 Gln 192 Ala 191 Gln 189 His 164 His 41 Cys 145 His 41 Met 165 Met 165	Moxifloxacin	Carboxylate Carboxylate Pyridone Carboxylate Methoxyl Fluorine Piperidine ring Piperidine ring Piperidine ring Pyridone ring Benzene ring	Conventional hydrogen bond Conventional hydrogen bond Conventional hydrogen bond Carbon hydrogen bond Carbon hydrogen bond Dipole–dipole π–cation Alkyl–alkyl π–alkyl π–alkyl π–alkyl	2.38 2.82 2.15 3.02 2.35 3.52 4.80 4.37 5.05 5.04 4.31
	Arg 188 Pro 168 His 163 Leu 167 Pro 168 Cys 145 His 163 Met 49 Met 165 Met 165	Chloroquine	Pyridine Chlorine Ethyl Chlorine Chlorine Ethyl Ethyl Pyridine ring Pyridine ring Benzene ring	Conventional hydrogen bond Carbon hydrogen bond Carbon hydrogen bond Alkyl Alkyl Alkyl π–alkyl π–alkyl π–alkyl π–alkyl	3.00 2.56 2.55 4.55 4.51 4.51 4.03 5.14 3.94 4.28
	Cys 145 Gln 189 His 164 Cys 145 His 41 Met 165 Leu 167 Pro 168	Nelfinavir	Sulfur Amide Propyl Phenyl ring Amide Cyclohexane ring Cyclohexane ring Cyclohexane ring	Conventional hydrogen bond Conventional hydrogen bond Carbon hydrogen bond π–sulfur π–lone pair Alkyl–alkyl Alkyl–alkyl Alkyl–alkyl	2.52 2.04 2.63 4.76 2.96 4.27 5.28 5.38

Figures 3d and 4d present the possible interaction of moxifloxacin inside the binding pocket of COVID-19 M^pro^ after 2D analysis in the Discovery Studio Visualizer. Corresponding amino acids that are significantly involved in the hydrophobic interactions are as follows: **His 164** (dipole–dipole) Ala 191, Gln 189 (carbon–hydrogen bond); Cys 145 (alkyl); **His 41**, Met 165, Met 165 (π–alkyl), **His 41** (cation–π); and 9 amino acids (Van der Waals). Strong hydrogen bond interaction between Gln 192 and carboxylate group and carbonyl oxygen atom of pyridone moiety increase the stability of the ligand–receptor complex (Table 2).

**Fig. 5:**
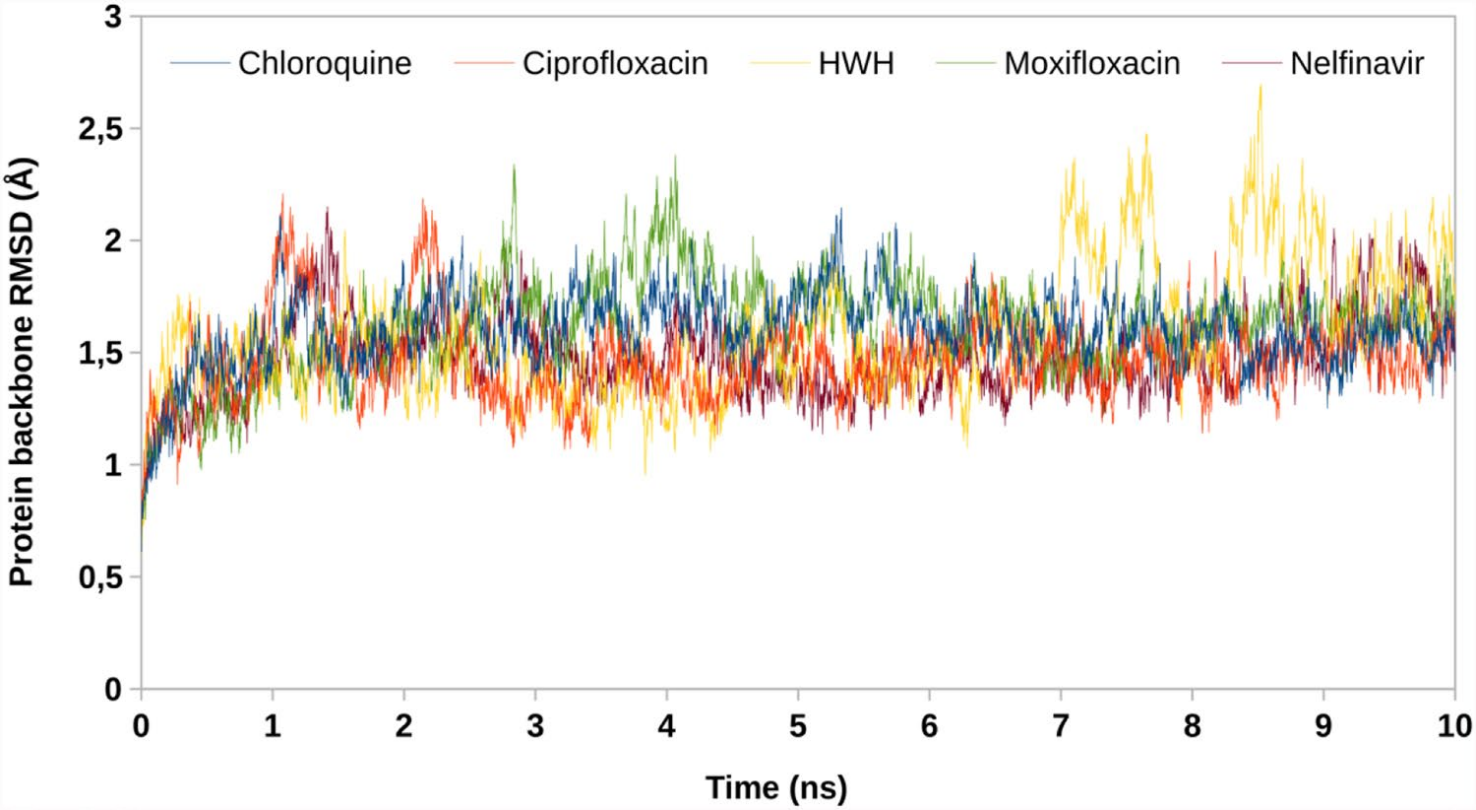
RMSD values of protein backbones in protein–ligand complexes

**Fig. 6:**
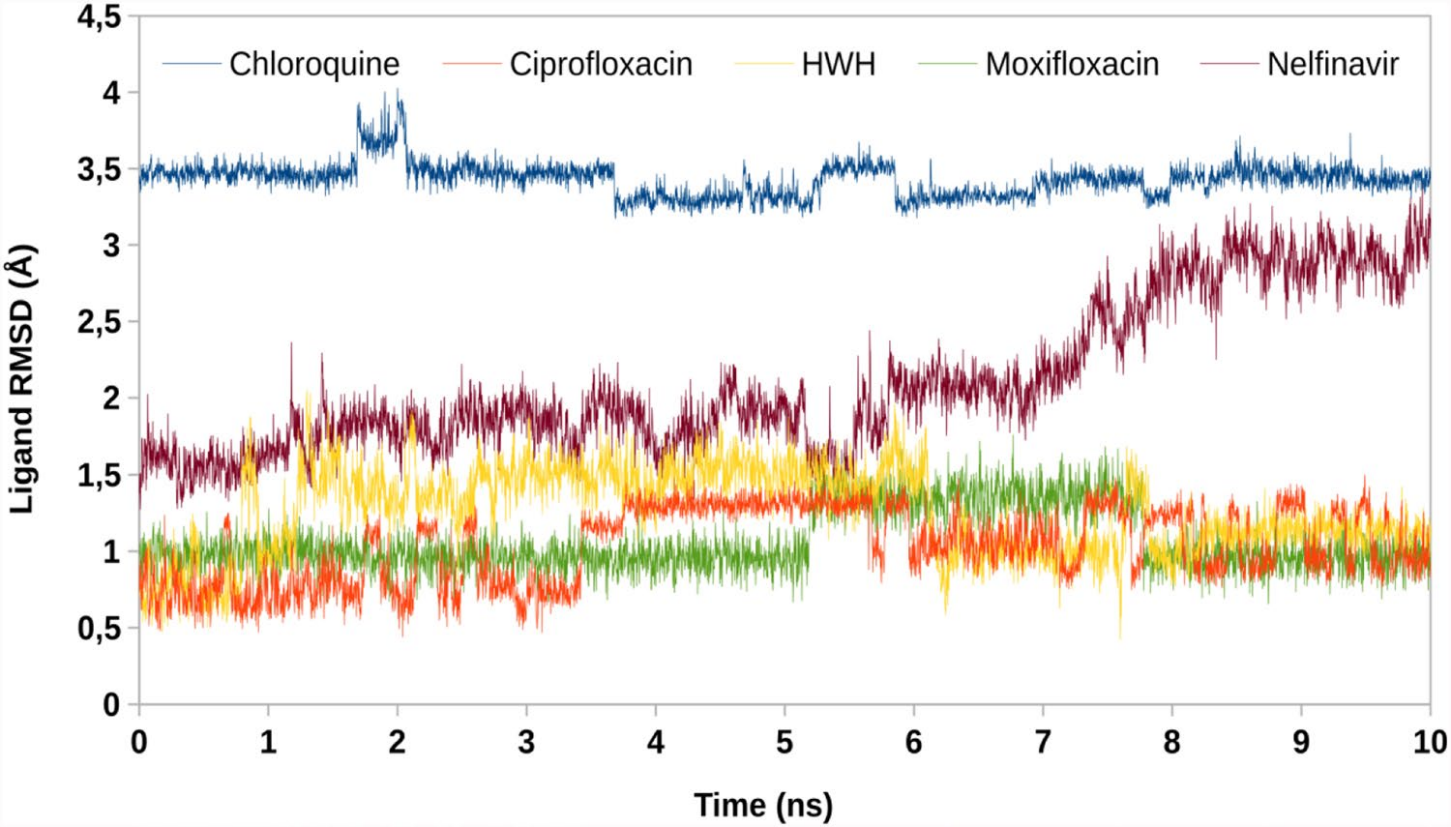
RMSD values of ligands in protein–ligand complexes

Complex of chloroquine with 5R7Z revealed that the endocyclic nitrogen atom of pyridine ring forms a hydrogen bond with Arg 188 (Figs. 3e, 4e). Dipolar interaction between chlorine atom and Leu 167 is also visible, as well as hydrophobic interaction involving an aromatic or aliphatic carbon in the receptor as well as an aliphatic or aromatic carbon in the ligand (including π–alkyl interaction of Met 49). Subsequent weak hydrogen bond interaction between Pro 168 and chlorine atom increase the stability of the ligand–receptor complex (Figs. 5, 6).

The analyses of the complex of nelfinavir and 5R7Z included calculations, distance measurements, and pose geometries that determined hydrogen bonding interactions of the ligand pose with Cys 145 and Gln 189. Moreover, Cys 145 forms another interactions between a sulfur atom and benzene ring (sulfur–π), His 164 forms interaction with propyl chain and (carbon hydrogen bond), and His 41 forms interaction with amide group (π–lone pair). In addition, numerous hydrophobic interactions between cyclohexyl ring of perhydroisoquinoline moiety (alkyl–π) influence the increase in the stability of the complex.

To verify stability of obtained docking poses, a molecular dynamics simulations were performed. Root mean square deviation (RMSD) of protein backbone and ligand in protein–ligand systems has been obtained. Low value of RMSD proves minor conformational changes of initial poses and validates docking protocol. Mpro demonstrated constant value of RMSD in all complexes, confirming reaching structural equilibrium. Ciprofloxacin, moxifloxacin and HWH have most optimal RMSD profile with medium value below 1.5 Å. On the other hand, nelfinavir during the first 7 ns of simulation keeps constant value, but next it quickly raises RMSD to value of 3 Å, similar to chloroquine (3.5 Å). These results indicate that compounds possessing highest Dock score (nelfinavir and chloroquine) may by less stable than lower-scoring compounds. Overall, molecular dynamics simulations show that ciprofloxacin and moxifloxacin are good anti-SARS-CoV-2 drug candidates.

## Discussion

To the best of our knowledge, there is currently no specific medicine or treatment for diseases caused by SARS-CoV-2 (2019-nCoV). Recently, the virus main protease (Mpro), also known as 3-chymotrypsin-like protease (3CLpro), has been successfully crystallised. The 3CLpro is automatically cleaved from poly-proteins to produce mature enzymes, and then further cleaves downstream non-structural proteins (Nsps) to release Nsp4–Nsp16, including the RNA-dependent RNA polymerase and helicase [25]. Since 3CLpro mediates the maturation of Nsps, which is essential in the life cycle of the virus, the inhibition of Mpro would prevent SARS-CoV-2 from replication and may constitute the potential drug target.

Finding new applications for already approved drugs with well-established pharmacokinetic and safety profile is more economical as well as much faster than developing a new drug and may consist of effective therapy strategy to overcome diseases. In the current study, we revealed the potential capacity of ciprofloxacin and moxifloxacin, members of fluoroquinolone broad-spectrum synthetic antibiotics, for binding with COVID-19 Main Protease (M^pro^), indicating the basis for a possible new strategy of COVID-19 treatment and ciprofloxacin and moxifloxacin repositioning to treat SARS-CoV-2 infections.

The detailed analysis of the ligand–protein interactions indicates that ciprofloxacin and moxifloxacin show a higher number of protein interactions than chloroquine and nelfinavir. It is worth emphasizing that ciprofloxacin binds to the protein with four strong hydrogen bonds and a significant number of hydrophobic interactions. Moreover, analysis of the docking results presented in Table 1 shows that ciprofloxacin and moxifloxacin exert lower binding energy values compared to nelfinavir. In addition, ciprofloxacin and moxifloxacin have most optimal RMSD profile with medium value below 1.5 Å.

Thus, both fluoroquinolone antibiotics may be potential inhibitors of the tested protease. It should be noted that ciprofloxacin and moxifloxacin represent the class of synthetic antibiotics used to treat upper respiratory tract diseases and also in the case of bacterial infections in which the resistance to the treatment with β-lactam antibiotics and macrolides was developed [26]. Another advantage of fluoroquinolones in the analysed context is their high bioavailability and the large distribution volume. Based on the good pharmacokinetic properties, ciprofloxacin is able to achieve higher concentrations in the target tissues than in plasma, which provides the opportunity for its widely use in the treatment of the respiratory and urinary tract infections [27, 28]. For example, it was noticed that the concentration of the drug after oral administration may reach the value in the lung tissue up to seven times higher than in the serum [29]. Ciprofloxacin can be safely taken at higher oral doses (above 500 mg twice a day) as a long-term therapy and thus different dosage options can be considered [30]. Therefore, the possible dual-mode of action could be especially used in the broad range of anti-infective activities in patients with COVID-19.

Due to the fact, that further studies need to be conducted to elucidate the in vitro as well as in vivo efficacy of the tested fluoroquinolones that could strengthen findings reported in the present study, we want to share our results with scientists in anti-SARS-CoV-2 research as soon as possible.
